# Artificial intelligence in neuroradiology: a scoping review of some ethical challenges

**DOI:** 10.3389/fradi.2023.1149461

**Published:** 2023-05-15

**Authors:** Pegah Khosravi, Mark Schweitzer

**Affiliations:** ^1^ Department of Biological Sciences, New York City College of Technology, CUNY, New York City, NY, United States; ^2^ Office of the Vice President for Health Affairs Office of the Vice President, Wayne State University, Detroit, MI, United States

**Keywords:** artificial intelligence, data privacy, deep learning, liability, machine learning, neural network, neuroradiology

## Abstract

Artificial intelligence (AI) has great potential to increase accuracy and efficiency in many aspects of neuroradiology. It provides substantial opportunities for insights into brain pathophysiology, developing models to determine treatment decisions, and improving current prognostication as well as diagnostic algorithms. Concurrently, the autonomous use of AI models introduces ethical challenges regarding the scope of informed consent, risks associated with data privacy and protection, potential database biases, as well as responsibility and liability that might potentially arise. In this manuscript, we will first provide a brief overview of AI methods used in neuroradiology and segue into key methodological and ethical challenges. Specifically, we discuss the ethical principles affected by AI approaches to human neuroscience and provisions that might be imposed in this domain to ensure that the benefits of AI frameworks remain in alignment with ethics in research and healthcare in the future.

## Introduction

Artificial intelligence (AI) leverages software to digitally simulate the problem-solving and decision-making competencies of human intelligence, minimize subjective interference, and potentially outperform human vision in determining the solution to specific problems.

Over the past few decades, neuroscience and AI have become to some degree, sublimated with the development of machine learning (ML) models using the brain circuits as the model for the invention of intelligent artifacts (1).

Neuroscience inspired and then ironically, validated the architecture of various AI algorithms (2) such as artificial neural networks (ANNs). This is a subset of ML approaches and is composed of units that are called artificial neurons which are typically organized into input, hidden, and output layers. One of the most successful ANN-based computational models is termed deep neural networks (DNNs) which consist of multiple hidden layers to learn more informative features and ubiquitous fields, by employing intelligence without explicit programming, to solve predictive problems such as segmentation and classification (3, 4).

Traditional, clinical neuroradiologists identify abnormalities of the spine, head and neck, and spinal cord through pattern abnormalities on MRI and CT (and occasionally other modalities). Radiomics and AI leverage imperceptible variations in images. Some have termed this as seeing the unseen. Research on applying AI to neuroradiology, and all imaging, has rapidly grown over the past decade. The number of scholarly publications related to the development and application of AI to the brain has more recently increased astoundingly (5).

The immense increase in publications on the development of AI models shows that AI is rapidly gaining importance in neuroradiologic research and clinical care. The growth in research is related to the combination of more powerful computing resources as well as more advanced measurement techniques (6–8), combined with advances in imaging sequences. Just a few somewhat basic examples of the application of AI models in neuroradiology include image reconstruction (9), improved image quality (10), lesion segmentation (11), specific identification of hemorrhages (12) and other lesions, as well as patterns in psychiatric disease (depression and schizophrenia) and neurologic disorders (such as Huntington's) (13), among many others. The more recent works on neuroimaging AI have concentrated on prognostication and greater personalization of treatments.

**Figure 1:**
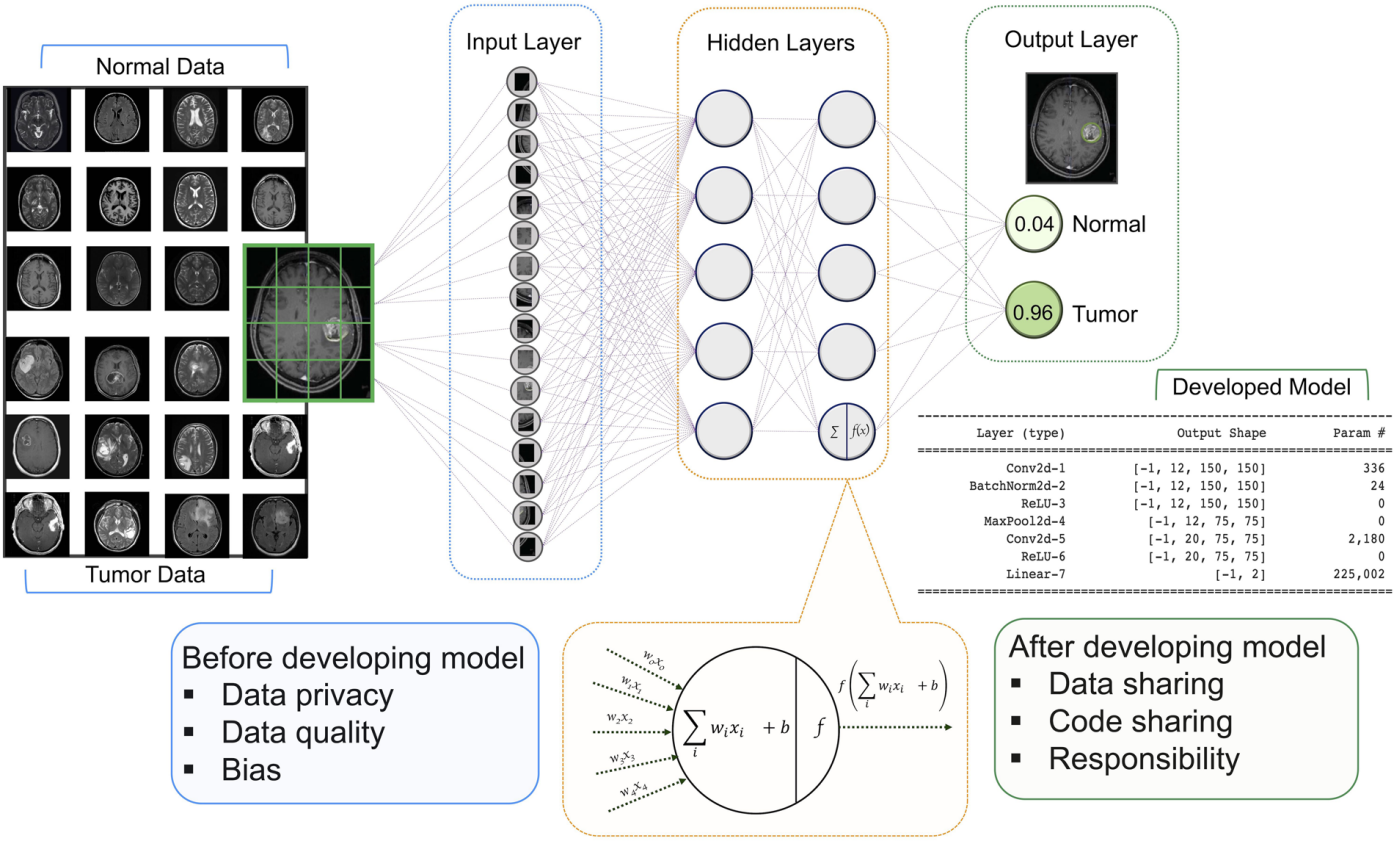
Architecture of a DNN with input, hidden, and two output layers. All layers are fully connected including multiple neurons. The goal of this network is to classify MR images into two classes of diagnoses (normal and tumor). Multiple images are broken down into their essential voxels and fed to the network as inputs. At the bottom is a zoomed-in view of an individual neuron in the second hidden layers (this architecture has two hidden layers with five neurons in each layer) including the summation function that binds inputs (features of image voxel) and the weights (values attached to features) together and the activation function along with the bias introduces non-linearity in the model. The final output is the values that show the probabilities of the two classification states. Neuroradiology images were obtained from the Kaggle link (15).

For neuroradiology, a deep learning (DL) model receives image series in the input layer, then the extracted features are analyzed in the hidden layer using various mathematical functions. The output layer encodes the desired outcomes or labeled states (e.g., tumor or normal). The goal of training a DL model is to optimize the network weights so that when a new series of sample images are fed to the trained model as inputs, the probabilities measured at the output are heavily skewed to the correct class (14) (Figure 1).

For the short term, these automatic frameworks will likely serve as decision-support tools that will augment the accuracy and efficiency of neuroradiologists. However, the progress in developing these models has not corresponded with progress in implementation in the clinic (16). This may be related to regulatory, reimbursement issues and perhaps most importantly concerns over the adjudication of liability (17). Relatedly, the implementation of AI methods leads to systemic risks with potentially disastrous consequences and societal implications (16).

In this paper, we contribute to the ethical framework of AI in neuroradiology. Below we present several specific ethical risks of AI, as well as air some principles that might guide its development in a more sustainable and transparent way. In our review, we highlight the ethical challenges that are raised by providing input for the AI models and the output of the established frameworks in neuroradiology (Figure 1). In each section, we also present the strategies that might be useful to tackle these challenges in the future.

## The profit of data

The value of any recorded medical observation - whether it is images, physical examination results, or laboratory findings - primarily lies in how they contribute to that patient's care. However, when the data are collected (and anonymized), this data can also be used to generate useful information for potential research. This information also may eventually have commercial potential. Databases can help us understand disease processes or may generate new diagnostic or treatment algorithms. These databases can be used to assess therapies in silico.

Because of the value of medical data, especially images, when conglomerated, those who participate in the health care system have an interest in advocating for their use for the most beneficial purposes. Hence, the controversy regarding who has the right to control and benefit from collected images, patients, or provider organizations.

There has been a paradigm shift over the last decade with respect to the ethos of data ownership (18). While some authors have advocated for researchers' right to access data (19), others have highlighted embedding trustworthiness into data-sharing efforts for all who participate in this process and benefit from it, including patients, providers, healthcare organizations, and industry (20, 21).

Most AI scientists believe in the ethical responsibility of all patients to share their data to improve patient care, now, and in the future. Thus, these researchers believe the data should be widely available and treated as a form of public good to be used for the benefit of future patients (21, 22). Individuals, in various countries and institutions, can and should have the right to opt out of anonymized data use. However, if that becomes widespread, it worsens another AI ethical issue-database biases (vide infra).

## Protection of data privacy

The significance of the diligent and ethical use of human data has been highlighted recently to promote a culture of data sharing for the benefit of the greater population, while also protecting the privacy of individuals (18). Therefore, before medical images can be used for the development of research or a commercial AI algorithm, they are required, in most jurisdictions, to obtain approval from the local ethical committee. An institutional review board (IRB) needs to assess the risks and benefits of the study to the patients. In many cases, existing (retrospective) data is used. Because the patients in this type of study do not need to undergo any additional procedures, explicit (written) informed consent is generally waived. With clinical trials, each primary investigator may need to provide approval to share data on their participants. In the case of a prospective study, where study data are gathered prospectively, written informed consent is necessary. After ethical approval, relevant data needs to be accessed, queried, and deidentified for all the health information (PHI) to meet the health insurance portability and accountability act (HIPAA) requirements in the United States or general data protection regulation (GDPR) in Europe, as well as securely stored. Notably, in certain countries, no formal IRB approval is needed for retrospective data use, and in others, patients sign blanket consent when they enter the local medical system. Although this is legal in those environments, to these authors it creates its own ethical concerns but does decrease database biases.

Other issues specifically related to neuroradiology, such as the presence of personal identifiers, which need to be removed both from the digital imaging and communications in medicine (DICOM), metadata, as well as from the images prior to data sharing (23). For example, surface reconstruction of volume acquisitions of the face and brain may allow re-identification by providing detailed images of the patient and generalization of facial recognition techniques (24). To prevent re-identification, defacing or skull-stripping techniques must be used in multicenter studies of neuroradiology (25, 26). Also, it is important to remove or blur the surface-based features in the high-resolution MR images to reduce the possibility of re-identification of individuals based on their surface anatomy through pre-processing algorithms. Furthermore, recent studies have shown that the generative adversarial networks (GANs) models and their synthetically generated data can be used to infer the training set membership by an adversary who has access to the entire dataset and some auxiliary information (27). These are somewhat unique issues in neuroimaging research. In this regard, there are software packages available that are able to remove the facial outline from high-resolution radiology images without removing or altering brain tissue (28, 29). Also, a new GAN architecture (privGAN) has been developed to defend membership inference attacks by preventing memorization of the training set to provide protection against this mode of attack (27).

Regarding the increasing usage of digital data in neuroscience, it is important to manage data resources credibly and reliably from the beginning. Data governance is the set of fundamental and policies that illuminate how an organization manages the data assets (30). Data governance is a pivotal module in the analysis of neuroradiology data and includes data stewardship, ownership, policies, and standards (30). The important underlying principle of data governance is the sense that those who manage patient data have an ethical responsibility to manage the data as trustee for the patients profit, the institution, and the public. Therefore, understanding data governance principles is critical in enabling healthcare professionals to adopt AI technologies effectively and avoid progress of these techniques due to concerns around data security and privacy (31).

Centralized and distributed networks are two organized methods that have been developed for tactical governance of data sharing (32). While in the centralized network the data such as repositories of neuroradiology images from multiple sites are aggregate into single central database, the distributed network model limits the flow of patient data to control the use of information and adhere to applicable legal regulations (32). While the former method improves the consistency of the data, enabling data access is more complex since it needs to adhere to the legal statutes of several organizations. The latter method enables users encounter less barriers related to participant willingness to share data, and legal obstacles. However, they struggle with harmonization of site-specific data before performing analyses at each site to make sure the data are consistent.

## Quality of annotated data

The quality and amount of the annotated images for training AI models are variable, based on the target task. Although using poor-quality images may lead to poor predictions and assessments, in heterogeneous large quantities, it is known that AI algorithms can be trained on relatively poor-quality images (23, 33). However, knowing the correct label for a given task to correlate with the imaging findings is a critical topic in policy documents and essential for the development of any AI system. In general, imaging data can be labeled in a variety of ways, including image annotations and segmentations. We have an ethical imperative to do AI research well, and one of the requirements is to have an adequate population, to reach conclusions from.

Most currently implemented AI algorithms for medical image classification tasks are based on a supervised learning approach. This means that before an AI algorithm can be trained and tested, the ground truth needs to be defined and attached to the image. The term ground truth typically refers to information acquired from direct observation [either of the images, or the patient (dermatology and surgical exploration), pathologic proof, or occasionally clinical follow-up]. For direct observation references standards images are annotated by medical experts- such as neuroradiologists.

Manual labeling is often used in the labeling and annotation of brain imaging data for AI applications. When a relatively small number of images are needed for AI development, medical expert labeling and segmentation may be feasible. However, this approach is time-consuming and costly for large populations, particularly for advanced modalities with numerous images per patient, such as CT, PET, or MRI. Prior to the widespread use of AI, and markedly improved through AI, semiautomated and automated algorithms have become somewhat widely used in labs.

Radiology reports are not created for the development of AI algorithms and the extracted information may contain noise. Although, recently more reports are structured, or protocol-based, more often they are narrative. These narrative reports have previously been assessed through natural language processing programs.

Neural networks can still be relatively robust when trained with noisy labels (34). However, one should be careful when using noisy labels for the development of clinically applicable algorithms because every labeling error could be translated to a decrease in algorithm accuracy. It is estimated that about 20 percent of radiology reports contain noticeable errors due to technical factors or radiologists' specific oversight or misinterpretations (35). Although errors or discrepancies in radiology are inevitable, some can be avoided by appropriate available strategies such as the fusion of radiological and pathological annotation, the use of structured reporting and computer-aided detection, defining quality metrics, and encouraging radiologists to contribute to the collation of these metrics (36).

AI could serve a role to reduce these errors and may help in the more accurate and time-efficient annotation of CT and MRI scans. However, this assumes a sufficiently sized dataset to adequately train the AI models. Furthermore, recent efforts in the automation of the annotation process particularly in neuroimaging data have shown a significant increase in the performance of the annotation process by using AI systems in large scale of data. For example, a study in brain MRI tumor detection indicated that applying semi-supervised learning to mined image annotations can improve tumor detection performance significantly by achieving an F1 score of 0.95 (37).

There is a trend toward interactive collaboration between AI systems and clinical neuroradiologists. The ability to self-validate and learn from mistakes of AI systems makes the system able to recognize their errors and self-correct their own data sets. On the other hand, external validation by neuroradiologists, manually fine-tuning the AI system, that it has made an error, and allowing the AI system to update its algorithm to avoid errors is helpful for the improvement of these models. Such interactive collaboration annotations have been used effectively for labeling open-source data sets to improve annotation quality while saving radiologists time (38, 39).

## Collaboration of AI and neuroradiologists

Neuroradiology is overall the third most common imaging subspecialty (40), and this subspecialty already heavily utilizes computing and machine technologies. As AI frameworks progress, they can support neuroradiologists by increasing their accuracy and clinical efficiency. However, it is important to recognize that AI applications can also replace some aspects of a neuroradiologist's work and therefore neuroradiologists may have concerns about the progress of AI beyond the role of assistance and replace them. This brings to mind, wider societal concerns about the future role of AI and human work.

A recent review paper explored the application of AI in neuroradiology and identified the majority of these applications provide a supportive role to neuroradiologists rather than replacing their skills. For example, assessing quantitative information such as the volume of hemorrhagic stroke and automatically identifying and highlighting areas of interest such as large vessel occlusions on CT angiogram (41). AI can facilitate and assist neuroradiologists' workflow at several stages. It can extract relevant medical information autonomously and exclude findings not relevant to the investigation. AI can standardize every scan and reduce any artifacts. It can help neuroradiologists by identifying abnormalities that are the most time-sensitive decision and annotating multiple images and other related clinical information at the same time (42).

While these capabilities are very promising, AI systems are not without limitations. For an AI system's algorithm to accurately work, they require large datasets and accurate expert labeling that may not always be possible. Therefore, as Langlotz suggested (43), maybe the neuroradiologists who use AI will replace those who don’t keep up to date with AI technologies.

Despite the widespread application of AI, it is important that radiologists remain engaged with AI scientists to both understand the capabilities of existing methods and direct future research in an intelligent way that is supported by the sufficient clinical need to drive widespread adoption (14). An AI-neuroradiologist collaborative model will have high value in potentially improving patient outcomes.

The subject of explicability is crucial as AI models are still considered as black box, due to lack of clarity regarding the data transformations after various passages within the convolutional neural networks (CNNs) despite the mathematical and logical processes (44). Therefore, the close collaboration between neuroradiologists and AI scientists with different expertise aids the development of AI models. While neuroradiologists may annotate images and query the quality of datasets, AI scientists build the algorithms and frameworks. Neuroradiologists who are familiar with digital imaging and informatics have the potential to investigate the black box and accompany the development process, confirming the respect of standards. ​​This collaboration establishes trust and establishes criteria for validating the performance of the AI models. The result of collaboration between AI scientists and neuroradiologists highlights the variety of benefits including the interpretability of the output of the algorithm. Also, while the AI scientists become familiar with the image features from the neuroradiologists' point of view, radiologists are trained to understand how AI works and how to integrate it into practice, how to evaluate its performance as well as recognize the ethical matters involved. In this formal accreditation process, the endpoint is the evaluation of the performance of the AI frameworks, neuroradiologists learning and using the AI systems, and patients trusting the physician using AI tools.

There is insufficient practicality related to the final stages such as reporting and communication compared to the early stages of the AI application (e.g., image pre-processing) (41). This indicates the opportunities for AI scientists and neuroradiologists to develop AI systems in areas beyond image interpretation that can be helpful for communicating the results of medical procedures to patients, particularly in the case that such results could alter the choice of therapy. Patients can benefit from counterfactual recommender systems that learn unbiased policies from data and it can be applied prospectively to support physicians' and patients' management decisions. Using this system have great potential and can be possible in the future when annotated patient data are accessible at the scale (4). Future development of AI applications that integrate software platforms intended for automatic rapid imaging review and provide a communication platform and optimized workflow to multidisciplinary teams will undoubtedly play a key role in more rapid and efficient identification for therapy, resulting in better outcomes in neurological treatments.

## Availability of models and data

The emergence of using AI in neuroradiology research has highlighted the necessity of code sharing because these are key components to facilitating transparent and reproducible experiments in AI research (45). However, despite the recommendations by editorial board members of the radiological society of north america (RSNA) journals (46, 47), recent studies revealed that less than one-third of the articles share code and adequately documented methods (48, 49) and most articles with code sharing by radiographic subspecialty are in neuroradiology (49).

Although these low rates of code sharing and code documentation may be discouraging, it has shown that code sharing over time is an uphill journey (49). Nonetheless, there is room for improvement, which can be facilitated by journals and the peer-review process. For example, reproducible code sharing can be improved by radiology journals through mandatory code and documentation availability upon article submission, reproducibility checks during the peer-review process, and standardized publication of accompanying code repositories and model demos which lead to faster and more collaborative scientific innovation.

In addition to code sharing, data availability is another key component of the reproducibility of AI research studies, because DL models may have variable performance on different neuroimaging datasets (50). However, the rate of data availability is even less than code-sharing (less than one-sixth of studies) (49) due to the challenges related to medical data sharing that we discussed earlier in this review. The latter study showed that the majority of studies that provided data used data from open-source datasets (51) such as TCIA which highlights the importance of these publicly released datasets to research (49). Code and data availability are ethical imperatives for AI research.

## Equal distribution of AI

Medical imaging, including MRI, is one of the most common ways of brain tumor detection. MRI scans such as T2-weighted and post-contrast T1-weighted are preferred since they provide more precise images and provide better visualization of soft tissue, therefore they can be used for brain tumor segmentation (52). CNNs, a class of DNNs, are used as prominent methods for medical image analysis. Therefore, neuroradiologists have become interested in obtaining image features and detecting brain tumors using CNNs to devote less time to screening medical images and more time to image analysis. Using AI tools by neuroradiologists at “the top of their license” is the most ethical construct. Hence, the use of AI to “flag” images, allows highly trained physicians to do what they do best, and what AI might not do best (for now).

Bias is an important ethical theme in research and clinical care. It is quite easy for potential bias to be embedded within algorithms that grow from “selected” data that are used to train algorithms (53, 54). Most research is performed at academic centers, more of this at the most prestigious academic centers. These centers see a biased population that skews towards affluence, education, and often disproportionately small numbers of patients of color. If algorithms are developed on datasets that are under- or over-representative of certain population subgroups, they may exhibit bias when deployed in clinical practice, leading to unequal access to care and potential harm to patients. Using biased data sets not only can potentially cause systemic inequities based on race, gender, and other demographic characteristics, but they may also limit the performance of AI as a diagnostic and treatment tool due to the lack of generalizability (54, 55).

A recently published review by Das et al. (56) investigated the risk of bias in AI data and methods that are used in brain tumor segmentation. They showed variance in designing the AI architecture and input data types in medical imaging increase the risk of bias. In another study to identify skin cancer from images, the researchers trained a model on a dataset including 129,450 samples, but less than 5% of these images were associated with dark-skinned individuals, thus the performance of the classifier could vary significantly across different populations (57). Furthermore, Larrazabal et al. (58) highlighted the importance of gender balance in medical imaging datasets for training AI systems in computational diagnosis. Using multiple DNN architectures and available image datasets, they showed dramatic changes in performance for underrepresented genders when balance and diversity are not fulfilled.

Some studies suggested using AI itself to potentially mitigate existing bias by reducing human error and biases that are present within healthcare research and databases (59). These studies addressed the issue of bias including building AI systems to reflect current ethical healthcare standards and ensuring a multidisciplinary approach to the deployment of AI (53, 60). To ensure that AI systems are fair and equitable, it is essential to address this challenge by developing algorithms that are trained on diverse datasets, including data from underrepresented populations. Furthermore, it is important to continually monitor AI systems to detect and mitigate any potential biases that may emerge over time. By addressing these challenges, we can help ensure that AI systems in neuroradiology are developed and deployed in a responsible and ethical manner and that they ultimately benefit more patients.

## Liability of the developed AI methods

In this section, we explore a frequent question regarding the application of AI in neuroradiology as to who is responsible for errors that may happen through the process of developing and deploying AI technology. AI algorithms may be susceptible to differences in imaging protocols and variations in patient numbers and characteristics. Thus, there are by the nature of the beast, specific scenarios where these algorithms are less reliable. Therefore, transparent communication about selection criteria and code sharing is required to validate the developed model by external datasets to ensure the generalizability of algorithms in different centers or settings (61).

Despite the success and progress of AI methods, they are ultimately implemented by humans, hence, some consideration of user confidence and trust (62) is important. Also, if AI systems were to fail - as is inevitable - especially if they are involved in medical decision-making, we should be able to determine why and how they failed. Hence, AI processes need to be auditable by authorities, thus enabling legal liability to be assigned to an accountable body (63). The transparency of AI applications in neuroradiology should be considered to ensure that responsibility and accountability remain with human designers or operators (64). To which degree, is liable, becomes an urgent and necessary question.

Several studies recommend that since healthcare professionals are legally and professionally responsible for making decisions in the patient's health interests, they should be considered responsible for the errors of AI in the healthcare setting, particularly with regard to errors in diagnostic and treatment decisions (65, 66). However, other studies emphasize that it is AI developers' responsibility to ensure the quality of AI technologies, including safety and effectiveness (67, 68). Although a small number of articles suggested commercial strategies for responsible innovation (69), the question is still debatable because AI processes are often complicated to understand and examine the output of AI systems (70).

The use of AI technology in neuroradiology should be cognizant that ultimately it is a person or persons who are responsible for the proper implementation of AI. Part of this responsibility lies in the appropriate implementation and use of guidelines to minimize both medical errors and liability. Guidelines should be implemented to reduce risks and provide reasonable assurance including the well-documented developed methods, research protocols, appropriate large datasets, performance testing, annotation and labeling, user training, and limitations (71). Especially since adopting AI in neuroradiology is increasing and it has enormous potential benefits, implementation of AI in this field requires thoughtful planning and diligent reassessment.

## Case numbers

When we do research, we have a moral obligation to provide the highest quality product we can. That is why IRBs not only look at the risks and benefits but also the research protocol, to ensure that the results will be worthwhile. One of the aspects of this IRB assessment is the size and type of population studied. Similarly in AI research, the database should include a large enough number of subjects to avoid overfitting. In addition, an external validation set is ethically required, to ensure generalizability. The numbers needed in the development set have been a moving target but are appropriately moving towards substantive requirements.

Dataset size is a major driver of bias and is particularly associated with the size of training data; the AI models should be trained on large, heterogenous, annotated data sets (72). Small training brain image datasets lead to overfitting in CNN models which causes diminishing robustness of developed methods due to data-induced bias.

Previous studies also have shown that using a large dataset decreases model bias and yields optimal performance because each MRI technique has different characteristics, therefore, integrating various modalities and techniques yields more accurate results than any single modality. Using multimodality and heterogenous datasets also can handle the overfitting problem regarding the trained model using a specific dataset to make the model generalize for an external validation set (33, 73).

In diseases, or neurologic disorders that are less common, finding large enough datasets may be difficult and may require pooled resources. When providing larger datasets is not possible due to rare diseases and under-represented populations, transfer learning, and data augmentation can be used to avoid overfitting due to small or limited data sets (74). The studies also presented some recommendations for improving the risk of bias in AI and providing a direction toward selecting appropriate AI attributes. They highlighted the role of some characteristics such as data size, gold standard, DL architecture, evaluation parameters, scientific validation, and clinical evaluation (56) in developing robust AI models. For instance, deep reinforcement learning and deep neuroevolution models have generalized well based on sparse data and successfully used for the evaluation of treatment response in brain metastasis and classification of brain tumors using MR images (75, 76). Nonetheless, for the time being, some assessments of unusual conditions may not be ethically researched and evaluated by AI, due to the limited availability of copious patient data.

## Context

Several ethical themes of the applications of AI in neuroradiology were presented in this review including privacy and quality of data that are used for training AI systems as well as the availability and liability of the developed AI models. In this scoping review, we addressed the ethics of AI within neuroradiology and reviewed overarching ethical concerns about privacy, profit, liability, and bias, each of which is interdependent and mutually reinforcing. Liability, for instance, is a noted concern when considering who is responsible for protecting patient privacy within data-sharing partnerships and for AI errors in patient diagnoses. We note that liability is related to specific laws and legislation, which by definition vary from one to another jurisdiction.

These broad ethical themes of privacy and security, liability, and bias have also been reported in other reviews on the application of AI in healthcare and radiology in general, and neuroradiology in particular. For example, in a review by Murphy et al. (69), the authors discussed ethical issues including responsibility surrounding AI in the field of health and pointed to a critical need for further research into the ethical implications of AI within both global and public health.

In another study, the authors discussed ethical principles including accountability, validity, the risk of neuro-discrimination, and neuro-privacy that are affected by AI approaches to human neuroscience (6). These latter two terms likely will be increasingly part of our conversations going forward.

Another article with a focus on patient data and ownership covered key ethical challenges with recommendations towards a sustainable AI framework that can ensure the application of AI for radiology is molded into a benevolent rather than malevolent technology (17). The other recent studies highlighted the intersection of data sharing, privacy, and data ownership with specific examples regarding neuroimaging (18). It is therefore clear from all review articles that the ethical challenges in AI should be considered in relation to all people who participate in developing AI technology including neuroradiologists and AI scientists.

In conclusion, the ethical challenges surrounding the application of AI in neuroradiology are complex, and the value of AI in neuroradiology increases by interdisciplinary consideration of the societal and scientific ethics in which AI is being developed to promote more reliable outcomes and allow everyone equal access to the benefits of these promising technology. Issues of privacy, profit, bias, and liability have dominated the ethical discourse to date with regard to AI and health, and there will undoubtedly be more that arise. AI is being developed and implemented worldwide, and thus, a greater concentration of ethical research into AI is required for all applications amidst the tremendous potential that AI carries, it is important to ensure its development and implementation are ethical for everyone.
